# Catalytic subunits of the phosphatase calcineurin interact with NF-κB-inducing kinase (NIK) and attenuate NIK-dependent gene expression

**DOI:** 10.1038/srep10758

**Published:** 2015-06-01

**Authors:** Miho Shinzawa, Hiroyasu Konno, Junwen Qin, Nobuko Akiyama, Maki Miyauchi, Hiroyuki Ohashi, Etsuko Miyamoto-Sato, Hiroshi Yanagawa, Taishin Akiyama, Jun-ichiro Inoue

**Affiliations:** 1 Division of Cellular and Molecular Biology, The Institute of Medical Science, The University of Tokyo, Minato-ku, Tokyo, Japan; 2 Department of Developmental and Regenerative Biology, Key Laboratory for Regenerative Medicine, Ministry of Education and International Base of Collaboration for Science and Technology, Ministry of Science and Technology, Jinan University, Guangzhou, China; 3 Division of Interactome Medical Sciences, The Institute of Medical Science, The University of Tokyo, Minato-ku, Tokyo, Japan; 4 Division of Molecular Biology, Research Institute for Biomedical Sciences, Tokyo University of Science, Yamazaki, Noda-shi, Chiba, Japan; 5 Department of Biosciences and Informatics, Faculty of Science and Technology, Keio University, Yokohama, Japan

## Abstract

Nuclear factor (NF)-κB-inducing kinase (NIK) is a serine/threonine kinase that activates NF-κB pathways, thereby regulating a wide variety of immune systems. Aberrant NIK activation causes tumor malignancy, suggesting a requirement for precise regulation of NIK activity. To explore novel interacting proteins of NIK, we performed *in vitro* virus screening and identified the catalytic subunit Aα isoform of serine/threonine phosphatase calcineurin (CnAα) as a novel NIK-interacting protein. The interaction of NIK with CnAα in living cells was confirmed by co-immunoprecipitation. Calcineurin catalytic subunit Aβ isoform (CnAβ) also bound to NIK. Experiments using domain deletion mutants suggested that CnAα and CnAβ interact with both the kinase domain and C-terminal region of NIK. Moreover, the phosphatase domain of CnAα is responsible for the interaction with NIK. Intriguingly, we found that TRAF3, a critical regulator of NIK activity, also binds to CnAα and CnAβ. Depletion of CnAα and CnAβ significantly enhanced lymphotoxin-β receptor (LtβR)-mediated expression of the NIK-dependent gene *Spi-B* and activation of RelA and RelB, suggesting that CnAα and CnAβ attenuate NF-κB activation mediated by LtβR-NIK signaling. Overall, these findings suggest a possible role of CnAα and CnAβ in modifying NIK functions.

Members of the nuclear factor (NF)-κB family of transcription factors regulate gene expression required for various physiological processes such as immune responses, inflammation, development, and cell proliferation^1^,^2^. This family consists of five members, RelA, RelB, c-Rel, NF-κB1 (p50 and its precursor p105), and NF-κB2 (p52 and its precursor p100), and promotes transcription as hetero- or homo-dimers^3^. NF-κB is sequestered in the cytosol by binding to inhibitory proteins in unstimulated cells, and then translocate to the nucleus upon receiving various ligand signals. Translocation of NF-κB is mediated by two distinct intracellular signaling pathways, canonical and non-canonical NF-κB pathways^4^. The canonical NF-κB pathway requires the IκB kinase (IKK) complex including IKKα, IKKβ, and IKKγ and results in nuclear translocation of NF-κB dimers typically consisting of RelA and p50, which in turn up-regulate genes required for innate immune responses and cell survival. In contrast to the canonical NF-κB pathway, the non-canonical NF-κB pathway does not require IKKβ and IKKγ, while IKKα is essential for mediation of the signaling pathway. IKKα phosphorylates inhibitory protein p100 that preferentially binds to RelB. Phosphorylation of p100 is followed by partial degradation of p100 to p52. Consequently, the p52 and RelB heterodimer complex is translocated into the nucleus for transcriptional activation^5^.

NF-κB-inducing kinase (NIK) was originally identified as a serine/threonine kinase that activates the canonical NF-κB pathway^6^. However, later studies revealed an essential role of NIK in non-canonical NF-κB activation. NIK-deficient mice and alymphoplasia (*aly*) mice, which have a dysfunctional point mutation in the *Nik* gene, lack lymph nodes, Payer’s patches, and organized structures of the spleen and thymus^7^,^8^,^9^. These phenotypes are similar to those of RelB-deficient mice^10^. Moreover, ligand-dependent phosphorylation of IKKα and processing of p100 are abolished by the absence of functional NIK in mouse embryonic fibroblasts (MEFs)^11^. These data suggest that NIK is a critical activator of the non-canonical NF-κB pathway to activate RelB via phosphorylation of IKKα and subsequent partial degradation of p100. In addition to its physiological significance, deregulation of NIK activation is reportedly associated with the onset of multiple myeloma and inflammatory diseases^12^,^13^,^14^. Under these pathological conditions, canonical and non-canonical NF-κB pathways are constitutively activated by NIK. These findings suggest a biological significance of the precise regulation of NIK-dependent NF-κB activation.

Activation of NIK is controlled by its phosphorylation and proteasome-dependent degradation^15^. In unstimulated cells, NIK is recruited to a complex consisting of TNF receptor-associated factor (TRAF) 3, TRAF2, and cellular inhibitor of apoptosis 1 or 2 (cIAP1/2) ubiquitin ligase through binding to TRAF3. The TRAF3-TRAF2-cIAP1/2 complex induces polyubiquitination and subsequent proteasomal degradation of NIK in unstimulated cells^16^. As a result, the constitutive degradation limits the amount of NIK protein at biochemically undetectable level in unstimulated cells. Ligand stimulation of receptors triggers self-degradation of the TRAF3-TRAF2-cIAP1/2 complex, thereby leading to stabilization and accumulation of NIK. Accumulated NIK induces autophosphorylation of Thr-559, which is required for phosphorylation of downstream IKKα for signal transduction^17^. In addition, a recent study has revealed novel feedback inhibition of NIK activity by IKKα-mediated phosphorylation of NIK at Ser-809, Ser-812, and Ser-815, leading to destabilization of NIK protein^18^.

Calcineurin is a serine/threonine protein phosphatase including a catalytic subunit (CnA) and regulatory subunit (CnB), which participates in calcium ion-dependent signal transduction pathways^19^. Calcineurin activates nuclear factor of activated-T cells (NFAT) by dephosphorylation. Previous studies have elucidated the roles of calcineurin in NF-κB activation. Calcineurin enhances T-cell antigen receptor (TCR)-mediated NF-κB activation by regulating formation of the Carma1-Bcl10-Malt1 complex^20^,^21^. In contrast, inhibition of calcineurin in murine macrophages enhances the nuclear localization of RelA induced by Toll-like receptor (TLR) signaling. Thus, calcineurin is a positive regulator of TCR signaling and a negative regulator of TLR signaling. These findings suggest the involvement of calcineurin in the canonical NF-κB pathway. However, the role of calcineurin remains to be determined in the non-canonical NF-κB pathway.

In this study, we identified calcineurin catalytic subunit Aα and Aβ isoforms (CnAα and CnAβ, respectively) as novel NIK-interacting proteins. Small interfering (si)RNA-mediated depletion of CnAα and CnAβ (CnAα/β) enhanced nuclear translocation of RelA and RelB and expression of a NIK-dependent target gene, *Spi-B*. Thus, our data suggest that CnAα/β are negative regulators of NIK-mediated signaling.

## Results

### NIK binds to the catalytic subunits of calcineurin

**Figure 1:**
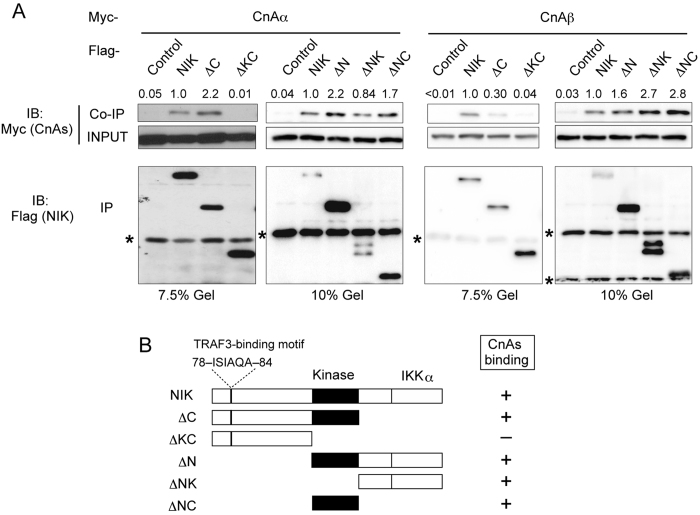
NIK interacts with CnAα/β through its kinase domain and C-terminal region. **A**. Co-immunoprecipitation of CnAα (left) and CnAβ (right) with NIK and its mutants (ΔC, ΔKC, ΔN, ΔNK, and ΔKC). NIK and its mutants expressed in cells are indicated at the top of panels. Control indicates the Flag-tagged expression vector. The upper panel (Co-IP) shows western blotting of immunoprecipitates using an anti-Flag antibody to detect co-immunoprecipitation of Myc-tagged CnAα or CnAβ. Band intensities of Co-IP bands relative to INPUT were normalized to that of full-length NIK and exhibited above the panel. The middle panel shows western blotting of total cell lysates using an anti-Myc antibody. The lower panels show western blotting of immunoprecipitates using the anti-Flag antibody to detect Flag-tagged NIK and mutants. Asterisks indicate bands of IgG chains used for immunoprecipitation. Results of one representative experiment of three are shown. Blots are cropped for clarity. Full-length blots of key data are presented in Supplementary Figure 2. **B**. Schematics of NIK and its deletion mutants used in this study. “Kinase” indicates the kinase domain. “IKKα” indicates the determined binding region of IKKα. A TRAF3-binding sequence is located in the N-terminal region. The Flag tag (abbreviated in this figure) was connected to the N-terminus of the wild-type protein and mutants. The binding ability of each protein for CnAα/β, as determined in Fig. 1A, is indicated at the right of each structure. “+” indicates positive for binding, and “−” indicates negative for binding.

To identify novel NIK-binding proteins, we performed *in vitro* selection of NIK-binding proteins using the combination of cell-free co-translation and an “*in vitro* virus” (IVV) technology^22^,^23^,^24^. This selection consisted of several steps: *in vitro* transcription and cell-free co-translation of bait NIK and prey cDNAs, IVV selection, and amplification of the selected IVVs by RT-PCR (see Methods for detail). Relatively weak interaction between NIK and NIK-binding peptides was detected by multiple rounds of this procedure. We screened a cDNA expression library from mouse embryonic thymus and obtained 29 candidates as novel NIK-binding proteins (Table 1). Because the function of NIK is positively or negatively controlled by phosphorylation and proteasome-dependent degradation^15^, respectively, we focused on possible regulators of these biochemical reactions (e.g., kinases, phosphatases, and ubiquitin ligases). Among the 29 candidates, we further validated CnAα as a possible regulator of NIK by co-immunoprecipitation studies (validation of some other candidates are shown in Table 1). To verify the interaction between CnAα and NIK in living cells, Flag-tagged NIK and Myc-tagged CnAα were transiently co-expressed in human embryonic kidney (HEK) 293T cells. A co-immunoprecipitation assay revealed that CnAα bound to NIK in HEK293T cells (Fig. 1A).

The CnA family consists of three isoforms encoded by different genes: CnAα, CnAβ, and the calcineurin catalytic subunit Aγ isoform (CnAγ). CnAα/β are expressed ubiquitously and usually function in a redundant manner, whereas expression of CnAγ is testis specific^25^. Despite the similarity in structure, the NIK-CnAβ interaction was not detected in the first screening, which could occur possibly due to technical reasons (e.g. possible biased amplifications during multiple rounds selections and PCR). Therefore, we tested binding of CnAβ to NIK in a co-immunoprecipitation assay. Indeed, co-immunoprecipitation indicated that CnAβ also interacted with NIK in HEK293T cells (Fig. 1A). These data suggested a common binding activity of CnAα/β for NIK. To gain some insight into the function of CnAα/β in NIK-dependent signaling, we next determined the responsible domains in NIK for its binding to CnAα/β.

NIK has a serine/threonine kinase domain that is essential for activation of NIK itself and downstream signal-transducing molecules^15^. The serine/threonine kinase region intervenes between the N-terminal and C-terminal regions (Fig. 1A). The N-terminal region contains the binding site for TRAF3 that is critical for degradation of NIK. The C-terminal region includes the binding site for IKKα that is phosphorylated by NIK and subsequently mediates downstream activation of the NF-κB pathway. To determine the CnAα/β-binding region in NIK, we analyzed various deletion mutants of NIK co-expressed with CnAα in HEK293T cells (Fig. 1A; left). A co-immunoprecipitation assay showed that deletion of both the C-terminal region and kinase domain (ΔKC mutant in Fig. 1B) abolished binding to CnAα, whereas the deletion mutant lacking only the C-terminal region still bound to CnAα (ΔC mutant in Fig. 1B). This finding suggests that the kinase domain binds to CnAα. Furthermore, the mutant lacking both the N-terminal region and kinase domain bound to CnAα (ΔNK in Fig. 1B), indicating that the C-terminal region also binds to CnAα. Thus, either the C-terminal region or the kinase domain (ΔNK and ΔNC in Fig. 1B, respectively) is sufficient for interacting with CnAα (Fig. 1A; right). As expected because of their similarity, binding regions of CnAβ in NIK were similar to those of CnAα (Fig. 1A) although the interaction of NIK with ΔC mutant of CnAβ is relatively weaker than that of CnAα. These data suggest that NIK recruits CnAα/β via two distinct regions, the kinase domain and C-terminal region.

**Figure 2:**
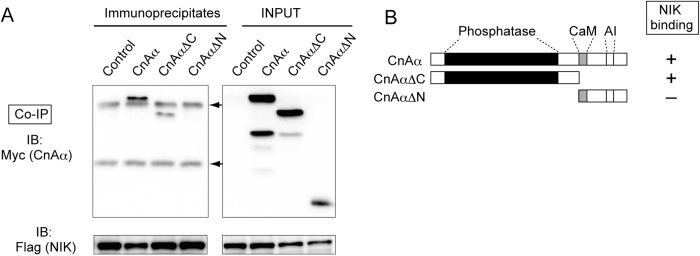
CnAα interacts with NIK through its phosphatase domain. **A**. Co-immunoprecipitation of CnAα and its mutants (ΔN and ΔCA) with NIK. CnAα and its mutants expressed in cells are indicated at the top of panels. Control indicates the Myc-tagged expression vector. The upper left panel (Co-IP) shows western blotting of immunoprecipitates using the anti-Flag antibody to detect co-immunoprecipitation of Myc-tagged CnAα and its mutants. The lower left panel shows western blotting of immunoprecipitates using the anti-Flag antibody to detect Flag-tagged NIK. The upper right panel shows western blotting of total cell lysates using the anti-Myc antibody. The lower right panels show western blotting of immunoprecipitates using the anti-Flag antibody to detect Flag-tagged NIK. Arrows indicate bands of IgG chains used for immunoprecipitation. Results of one representative experiment of three are shown. Blots are cropped for clarity. Full-length blots of key data are presented in Supplementary Figure 2. **B**. Schematics of CnAα and its deletion mutants used in this study. “Phosphatase” indicates the phosphatase domain containing the catalytic domain and regulatory subunit-binding domain. “CaM” indicates a potential calmodulin-binding domain. “AI” indicates the auto-inhibitory domain. The Flag tag (abbreviated in this figure) was connected to the N-terminus of the wild-type protein and mutants. The binding ability of each protein for NIK, as determined in Fig. 2A, is indicated at the right of each structure. “+” indicates positive for binding, and “−” indicates negative for binding.

We next examined the NIK-binding region in CnAα. CnAα consists of several domains: an N-terminal phosphatase catalytic domain, regulatory subunit binding domain, calmodulin-binding domain, and autoinhibitory domain (Fig. 2A)^24^. C- or N-terminal deletion mutants of CnAα (CnAα ΔC and CnAα ΔN in Fig. 2A) were co-expressed with NIK in HEK293T cells. A co-immunoprecipitation assay showed that NIK bound to the C-terminal deletion mutant (CnAα ΔC), but not the N-terminal deletion mutant (CnAα ΔN) (Fig. 2B). Thus, CnAα binds to NIK via its phosphatase domain.

These data suggest that the phosphatase domain of CnAα/β interacts with the kinase domain and C-terminal domain of NIK. Because NIK is recruited to a protein complex consisting of TRAF2, TRAF3, and cIAPs in unstimulated cells, we next determined whether CnAα/β also interact with this protein complex.

### CnAα/β bind to TRAF3

**Figure 3:**
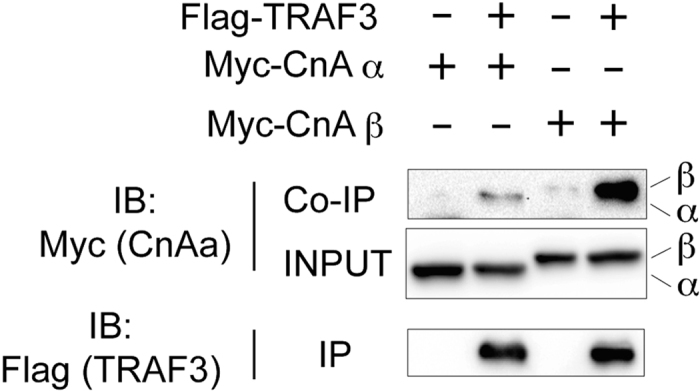
CnAα/β interact with TRAF3. Co-immunoprecipitation of CnAα/β with TRAF3. Combinations of proteins expressed in cells by transfection are indicated at the top. “−” indicates that Flag-tagged or Myc-tagged expression vectors were introduced by transfection. The upper panel (Co-IP) shows western blotting of immunoprecipitates using the anti-Myc antibody to detect co-immunoprecipitation of Myc-tagged CnAα or CnAβ. The middle panel shows western blotting of total cell lysates using the anti-Myc antibody. The lower panels show western blotting of immunoprecipitates using the anti-Flag antibody to detect Flag-tagged TRAF3. Results of one representative experiment of three are shown. Blots are cropped for clarity. Full-length blots of key data are presented in Supplementary Figure 2.

The protein complex consisting of TRAF2, TRAF3, and cIAP1 or cIAP2 mediates polyubiquitination of NIK, thereby initiating its degradation in unstimulated cells^5^. TRAF3 in this protein complex binds to NIK. Interestingly, a co-immunoprecipitation assay indicated that CnAα/β bound to TRAF3 in transfected HEK293T cells (Fig. 3). Thus, in addition to NIK, CnAα/β bind to TRAF3. These results support the idea that CnAα/β binds to a transient protein complex containing TRAF3 and NIK, which should be formed before proteasome-dependent constitutive degradation of NIK in unstimulated cells. Interestingly, affinity of CnAβ with TRAF3 seemed to be higher than that of CnAα, which implying the difference between these two homologues in contribution to function of NIK-TRAF3 complex. Because CnAα/β interact with NIK and its regulator TRAF3, we next addressed the roles of CnAα/β in NIK-mediated gene expression induced by receptor ligations.

### Transcription factor Spi-B is a target gene of NIK-mediated signaling triggered by ligation of lymphotoxin β-receptor

TNF receptor family lymphotoxin β receptor (LTβR) signaling has been reported to activate NIK-mediated non-canonical NF-κB activation and thereby inducing the expression of numerous chemokines including *Cxcl13*, *Ccl19*, and *Ccl21* in peripheral lymphoid tissues^26^,^27^,^28^. However, we failed to detect significant up-regulation of these genes in MEFs, which is consistent with previous observations^29^,^30^. Therefore, we first searched for a target gene induced by LTβR-NIK signaling in MEFs.

**Figure 4:**
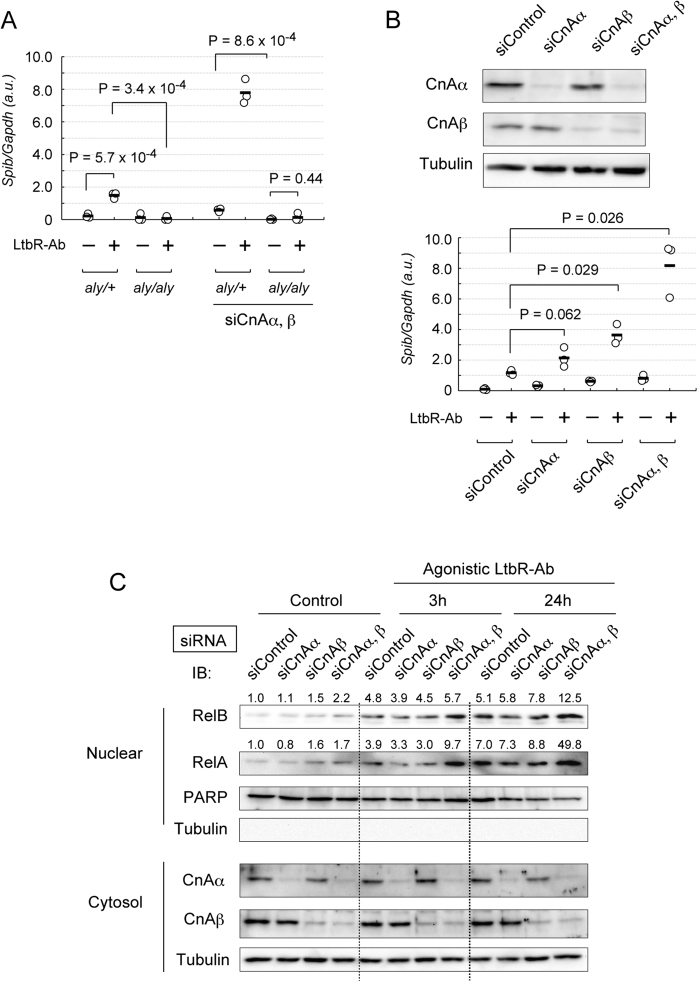
CnAα/β attenuate NIK-dependent Spi-B expression and nuclear translocation of NF-κBs. **A**. Quantitative RT-PCR analysis of *Spi-B1* expression in *aly/+* and *aly/aly* MEFs treated with an agonistic anti-LtβR antibody. *Aly/+* and *aly/aly* MEFs or *aly/+* and *aly/aly* MEFs depleted of both CnAα/β by siRNAs were stimulated with the agonistic anti-LtβR antibody. Expression of *Spi-B1* was evaluated by qPCR analysis. Representative data of three independent triplicate wells are shown. Black bars indicate mean values. *P* indicates the results of Student’s t-tests. **B**. qPCR analysis of *Spi-B* expression in MEFs depleted of CnAα, CnAβ, or both CnAα/β. Wild-type MEFs depleted of CnAα and/or CnAβ by siRNAs (upper panels) were stimulated with the agonistic anti-LtβR antibody. Representative data of three independent triplicate wells are shown. Black bars indicate mean values. *P* indicates the results of Student’s t-tests. Blots are cropped for clarity. Full-length blots are presented in Supplementary Figure 2. **C**. Depletion of CnAs enhances nuclear localization of RelA and RelB induced by LtβR signaling. CnAα-, CnAβ-, or CnAα/β-depleted MEFs were treated with the agonistic anti-LtβR antibody for 3 and 24 h (LtβR-Ab), or untreated (control). Nuclear and cytosolic protein fractions were analyzed by western blotting. Band intensities of RelA and RelB relative to PARP were normalized to that of control (control siRNA-treated cells without stimulation) and exhibited on the top of panels. Antibodies used for western blotting are indicated at the left of panels. Blots are cropped for clarity. Full-length blots are presented in Supplementary Figure 2.

We have recently found that NIK activation induces expression of a splice variant of Spi-B (hereafter referred to as *Spi-B1*) in TNF receptor family member RANK signaling^31^. That study suggested that *Spi-B1* is a direct target gene of NIK-mediated activation of NF-κB signaling because overexpression of NIK and the RelB complex activates the proximal promoter of the *Spi-B1* gene^31^. Because LTβR signaling activates NIK-dependent NF-κB pathways similarly to RANK signaling^32^, we first tested whether LtβR signaling induces *Spi-B1*. MEF cells were stimulated with an agonistic anti-LtβR antibody. Quantitative PCR (qPCR) analysis indicated that LtβR signaling efficiently up-regulated *Spi-B1* (Fig. 4A,B).

We next confirmed that LtβR signaling-mediated expression of *Spi-B1* is dependent on NIK activity. The *Aly/aly* mice line has a point mutation in the coding region of the *Nik* gene^8^. Because the *aly/aly* mutation abrogates binding of NIK to IKKα^33^, there is a severe impairment in NF-κB activation mediated by NIK-IKKα. We isolated MEFs from *aly/aly* mice and determined whether LtβR signaling-mediated *Spi-B1* expression is dependent on the NIK-IKKα axis by qPCR analysis. In fact, up-regulation of *Spi-B1* induced by LtβR stimulation was abolished in *aly/aly* MEFs (Fig. 4A). Thus, the NIK-IKKα interaction is essential for LtβR signaling-dependent expression of *Spi-B1* in MEFs.

Because the LtβR-NIK-IKKα signaling axis was confirmed to induce *Spi-B1* expression in MEFs, we next addressed the function of CnAα/β in the LtβR signaling-dependent *Spi-B1* expression in MEFs.

### CnAα/β attenuates expression of *Spi-B* and nuclear translocation of RelA and RelB induced by NIK-mediated signaling

Protein expression of CnAα/β was suppressed by siRNA-mediated knockdown in MEFs (Fig. 4B). We found that siRNA-mediated knockdown of CnAα/β resulted in a significant increase in the expression *Spi-B* induced by LTβR ligation (Fig. 4B, right). Effect of the CnAβ depletion were prominent as compared to that of the CnAα depletion, which is consistent with the observation that the affinity of CnAβ with TRAF3 was higher than that of CnAα (Fig. 3). Double knockdown of CnAα/β led to remarkable up-regulation of LtβR-mediated *Spi-B* expression, suggesting partial redundancy of these two isoforms. The enhancement of *Spi-B* expression by CnAα/β knockdown was not observed in *aly/aly* MEFs (Fig. 4A). This result is consistent with the idea that CnAα/β-dependent regulation of Spi-B expression is mediated by NIK. The basal level of *Spi-B* expression (without anti-LtβR antibody stimulation) seemed to be elevated by CnAα/β deletion (Fig. 4A,B).

**Figure 5:**
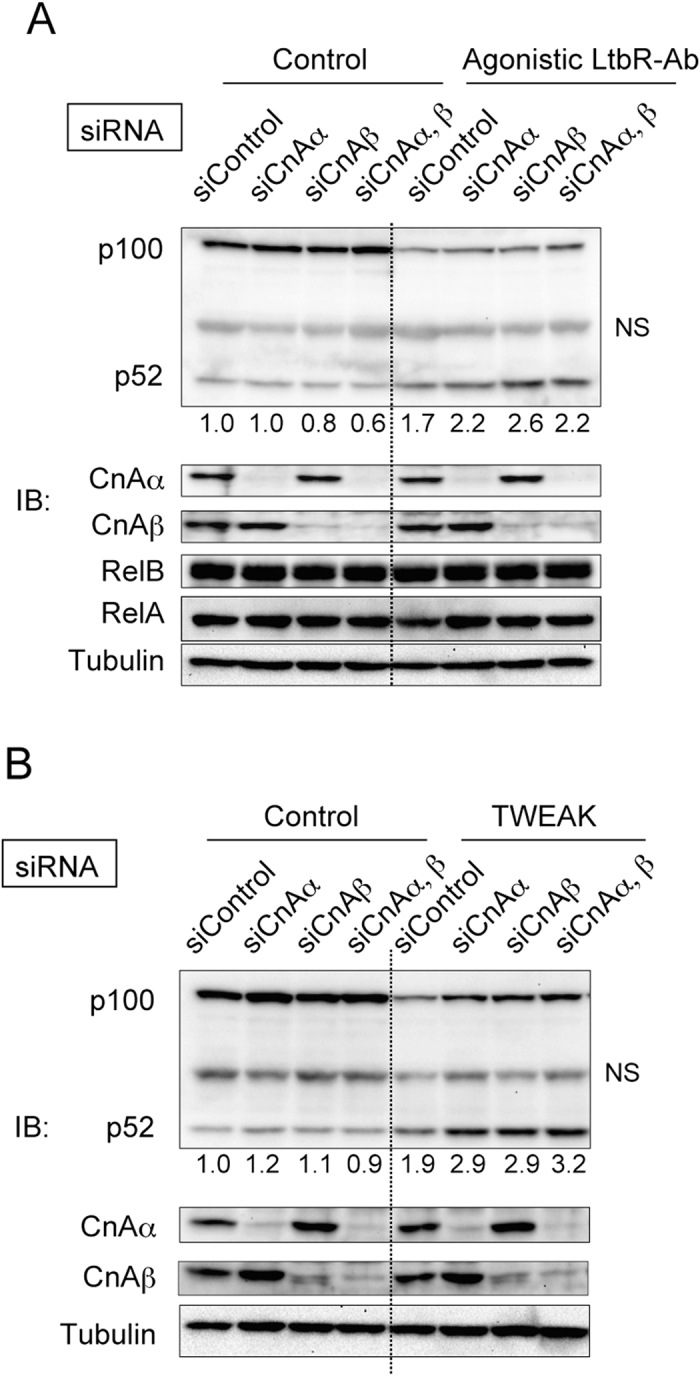
CnAs negatively regulate LtβR- and TWEAK-dependent processing of p100 to p52. **A.** Effect of CnAα/β depletion on LtβR-dependent processing of p100 to p52. Endogenous CnAα/β were depleted in MEF cells by siRNA-mediated knockdown. CnAα-, CnAβ-, or CnAα/β-depleted MEFs were treated with the agonistic anti-LtβR antibody for 24 h (LtβR-Ab) or untreated (control). Total cell lysates were analyzed by western blotting. siRNA used for knockdown are indicated at the top of panels. Antibodies used for western blotting are indicated at the left of panels. Band intensities of p52 relative to Tubulin were normalized to that of control (control siRNA-treated cells without stimulation) and exhibited under panels. Blots are cropped for clarity. Full-length blots are presented in Supplementary Figure 2. **B**. Western blotting of total lysates of MEFs treated with TWEAK. Endogenous CnAα/β were depleted in MEFs by siRNA-mediated knockdown. CnAα-, CnAβ-, or CnAα/β-depleted MEFs were treated with recombinant TWEAK for 3 h or untreated (control). Total cell lysates were analyzed by western blotting. siRNAs used for knockdown are indicated at the top of panels. Band intensities of p52 relative to Tubulin were normalized to that of control (control siRNA-treated cells without stimulation) and exhibited under panels. Antibodies used for western blotting are indicated at the left of panels. Blots are cropped for clarity. Full-length blots are presented in Supplementary Figure 2.

NIK-mediated activation of canonical and non-canonical NF-κB pathways leads to activation of RelA and RelB complexes, respectively, thereby enhancing gene expression^15^. Because CnAα/β negatively regulated NIK-mediated *Spi-B* expression, we next determined the role of CnAα/β in NF-κB activation induced by LtβR-NIK signaling. Because nuclear translocation is a critical hallmark of NF-κB activation, we examined whether CnAα/β negatively regulate LtβR signaling-mediated nuclear translocation of RelA and RelB. As reported previously^34^, nuclear RelA and RelB levels were increased by stimulation with the agonistic anti-LtβR antibody in MEFs. Depletion of both CnAα/β increased the amount of nuclear RelA and RelB induced by LtβR signaling (Fig. 4C), whereas the total amount of RelA and RelB was not significantly influenced by LtβR stimulation (Fig. 5A).

These data suggest that CnAα/β cooperatively attenuate NIK-mediated NF-κB activation, thereby negatively regulating expression of the NIK-dependent gene *Spi-B*. Therefore, we next determined whether CnAα/β is involved in the NIK-mediated signaling pathway of non-canonical NF-κB activation.

### CnAα/β negatively regulate processing of p100 to p52 induced by LtβR and tumor necrosis factor-like weak inducer of apoptosis (TWEAK) signaling

It is known that LtβR-NIK signaling induces processing of p100 to p52^5^. Indeed, stimulation with the agonistic anti-LtβR antibody led to a reduction of p100 and an incremental increase of p52 in MEFs (Fig. 5A). CnAβ depletion slightly increased the amount of p52 induced by stimulation with the anti-LtβR antibody (Fig. 5A). However, there were marginal effects of CnAα/β depletion. Therefore, we used recombinant TWEAK protein as a ligand to confirm the effect of CnAα/β depletion on p100 processing. Binding of TWEAK to its receptor, Fn14, effectively induced processing of p100 to p52 in MEFs (Fig. 5B), which is consistent with previous studies^35^,^36^. Depletion of CnAα or CnAβ caused an increase in the amount of processed p52. Interestingly, the level of total NF-κB2 protein (i.e., both p52 and p100) in cells was also increased in CnAα/β knockdown MEFs stimulated with TWEAK (Fig. 5B). Thus, CnAα/β inhibit the expression and processing of p100 induced by the TWEAK-Fn14 axis. Because canonical NF-κB activation reportedly up-regulates p100 expression^34^, these data are consistent with the idea that CnAα/β attenuates both canonical and non-canonical NF-κB activation. Our data suggest that CnAα/β negatively regulates processing of p100 to p52 induced by ligand signaling.

## Discussion

Calcium ions play a critical role in a variety of signal transduction pathways as a second messenger^37^. Calcineurin mediates certain calcium signaling pathways by dephosphorylation of NFAT^24^. Several studies have reported that intracellular calcium ions modulate NF-κB activity. Calcineurin enhances activation of the canonical NF-κB pathway in T cells by promotion of Carma1-Bcl10-Malt1 complex formation^20^,^21^, while it attenuates TLR-dependent activation of the canonical NF-κB pathway by inhibition of the essential adaptor MyD88 and TRIF^38^. Here, we propose that CnAα/β negatively regulate the non-canonical NF-κB pathway mediated by NIK. Thus, our data suggest the possibility of novel cross-talk between calcium signaling and the non-canonical NF-κB pathway induced by TNF family signaling.

An important aspect is the mechanism by which CnAα/β control NIK activity. Deletion mutant experiments suggest that CnAα/β interact with NIK via the phosphatase domain. Because NIK mediates downstream signaling by autophosphorylation and phosphorylation of downstream target molecules, it is possible that NIK-interacting CnAα/β dephosphorylates substrates of NIK, thereby inhibiting the function of NIK as a signal transducer. Further in-depth structural and biochemical studies are necessary to determine the molecular mechanism of CnAα/β-mediated regulation of NIK activity.

Single knockdown of CnAα or CnAβ enhanced processing of p100 to p52 induced by TWEAK signaling, whereas an additive effect was not observed by double knockdown of CnAα/β (Fig. 5). Assuming that the role of CnAα/β in regulation of NIK functions is redundant, NIK-dependent p100 processing may be already maximized by elimination of either CnAα or CnAβ. Conversely, nuclear localization of RelA and RelB was not clearly enhanced by single knockdown of CnAα or CnAβ, but it was increased by double knockdown of CnAα/β. Moreover, expression of the target *Spi-B* gene was more efficiently up-regulated in double knockdown cells compared with that in single knockdown cells. One possible explanation for these observations is that CnAs negatively regulate the NIK-mediated NF-κB activation pathway via two independent mechanisms. Thus, one mechanism influences processing of p100 to p52 and may be relatively sensitive to reductions in the amounts of CnAs in cells, while another mechanism affects nuclear localization of the NF-κB complex and may be less sensitive to CnA depletion. This idea may be consistent with the fact that CnAs bind to NIK at two distinct regions (Fig. 1). Thus, CnAs may inhibit the function of NIK via two mechanisms through interacting with the kinase domain or C-terminal region in NIK.

Deregulation of NF-κB induces tumorigenesis and inflammatory diseases^15^,^39^. Therefore, NF-κB activity needs to be finely tuned and ceased appropriately at the end of stimulation. Previous studies have indicated that deregulation of NIK leads to activation of canonical and non-canonical NF-κB pathways, which is associated with the pathogenesis of multiple myeloma^12^,^13^. Our data imply that CnAα/β may be novel modulators of NIK activity. Although it is unknown whether CnAα/β-mediated inhibition of NIK activity is also active in other cell types such as B cells or plasma cells, it would be interesting to investigate whether abolition or attenuation of calcineurin-mediated NIK inhibition can initiate or promote malignant B-cell tumors or other types of tumors.

Because proper regulation of NIK activation is essential to prevent the onset of cancer and inflammatory diseases, further studies on calcineurin-mediated inhibition of NIK activity might provide important insights into the development of anti-tumor or anti-inflammatory drugs in the future.

## Methods

### Ethics statement

All experiments using mice were approved by the Committee for Animal Experiments of the Institute of Medical Science, The University of Tokyo (approved number: H13-26), Mice were handled in accordance with the Guidelines for Animal Experiments of the Institute of Medical Science, The University of Tokyo.

### *In vitro* virus selection

First, randomly primed reverse transcription of fetal thymus poly(A)+ mRNAs were subjected to ligation mediated amplification and multi-step PCRs to create cDNA constructs for *in vitro* expression. The resulting PCR products (SP6-Ω-T7-Flagment-Kpn1-FLAG) were purified with a QIAquick PCR Purification Kit (Qiagen, Germany) and transcribed into mRNA with a RiboMAX Large Scale RNA Production System-SP6 (Promega, WI, USA) and an m7G(5’)ppp(5’)G RNA Cap Structure Analog (Ambion, Life Technologies, CA, USA). After purification of the transcribed mRNAs using an RNeasy 96 BioRobot 8000 Kit (Qiagen), PEG Puro spacer was ligated to the 3’ ends of mRNAs using T4 RNA ligase (Promega) and the RNA was purified again. A cDNA for the bait (NIK) was prepared similarly. *In vitro virus* selection was performed as previously reported. Briefly, mRNA templates used as bait and prey were co-translated in a wheat germ extract (Zoegene Corporation, now Molecuence Corporation) for 1 h at 26 °C in 96-well plates by using Qiagen Biorobot 8000. At the same time, the *in vitro virus* molecules were formed by covalently attaching the 3’ end of mRNA for prey to the C-terminus of its coding protein via puromycin. After each round of selection, prey mRNA was amplified by RT-PCR, followed by the *in vitro* transcription and translation reactions that prepared the library for the next round of selection. After four rounds of selection, interaction sequence tags obtained by *in vitro virus* selection were identified by Takara Bio Inc., Otsu, Japan and Shimadzu Corporation, Genomic Research Center, Kyoto, Japan. A mock experiment was performed without bait protein as the negative control to eliminate technical false positive results.

### Cell culture, transfection, and siRNA-mediated knockdown

*Aly/+* and *aly/aly* MEFs were prepared from whole embryos of *aly/+* and *aly/aly* mice (CLEA, Japan). Briefly, embryos were dispersed in PBS containing 0.25% trypsin and 1 mM EDTA. After removal of the enzyme solution, the dispersed cells were cultured in Dulbecco’s modified Eagle’s medium (DMEM) supplemented with 10% fetal bovine serum, glutamate, penicillin (100 U/ml), and streptomycin (100 U/ml). Attached cells were subjected to assays. HEK293T cells and MEFs were maintained in DMEM supplemented with 10% fetal bovine serum, glutamate, penicillin (100 U/ml), and streptomycin (100 U/ml). Transfection of HEK293T cells was performed using the calcium phosphate method. siRNAs were transfected using RNAiMAX reagents (Life Technologies, Rockville, MD). As a control siRNA, we used a medium GC % negative control Stealth siRNA (Invitrogen, Carlsbad, CA). The following double strand siRNAs (Life Technologies) were used to silence CnAα and CnAβ: CnAα, sense 5´-UAA ACG UGA AAU ACU CUG UGA GGU G-3′ and antisense 5′-CAC CUC ACA GAG UAU UUC ACG UUU A-3′; CnAβ, sense 5′-GCU GUG CAG CAA GAU GGU UUC AAU U-3′ and antisense 5′-AAU UGA AAC CAU CUU GCU GCA CAG C-3′.

### Plasmids

Expression vectors encoding full-length and truncated forms of NIK and CnAα were generated by PCR amplification of NIK and CnAα cDNAs (provided by RIKEN), followed by subcloning the amplified DNA fragments into vectors.

### Antibodies and reagents

We used the following antibodies: anti-Flag M2 (F3165) (Sigma-Aldrich, St Louis, MO), mouse anti-Myc (sc-40), rabbit anti-Myc (sc-789), mouse anti-HA (sc-805), anti-Parp1 (sc-25780), mouse anti-TRAF3 (sc-6933), rabbit anti-TRAF3 (sc-1828), anti-p65 (sc-8008) (Santa Cruz Biotechnology, Santa Cruz, CA), anti-NIK (4994), anti-p52 (4882), anti-RelB (4922s) (Cell Signaling, Beverly, MA), anti-CnAα (07-067), anti-CnAβ (07-068), anti-tubulin (CP06) (Millipore, Darmstadt, Germany). The following reagents used were in experiments: MG132 (Peptide Institute, Osaka, Japan) and an agonistic anti-LtβR antibody (Alexis Biochemicals, Läufelfingen, Switzerland).

*In vitro* virus selection was performed as reported previously^22^. Briefly, a cDNA library was prepared from mouse fetal thymus RNA (embryonic day 18.5). NIK mRNA was used as bait, and prey were co-translated in a wheat germ extract (Molecuence, Yokohama, Japan) using a Qiagen Biorobot 8000. After four rounds of selection, we identified interaction sequence tags obtained by *in vitro* virus and verified them as reported previously^23^,^40^.

### Immunoprecipitation and immunoblotting

Lysates of HEK293T cells and MEFs were prepared in TNE buffer (50 mM Tris, pH 7.4, 150 mM NaCl, 1 mM EDTA, 1% Nonidet P-40, 1 mM sodium orthovanadate, and a protease inhibitor mixture). The lysates were precleared in a protein G-sepharose column (GE Healthcare, Chalfont St Giles, UK) and immunoprecipitated with the indicated antibodies, followed by incubation with protein G-sepharose. For endogenous immunoprecipitation of TRAF3, MEFs were pretreated with 10 mM MG132 for 2 h before harvesting. For immunoblot analysis, immunoprecipitates or cell extracts were eluted with SDS loading buffer (67.5 mM Tris-HCl, pH 6.8, 2.25% SDS, 10% glycerol, 5% β-mercaptoethanol, and bromophenol blue) and resolved by SDS-polyacrylamide gel electrophoresis. The proteins were transferred to polyvinylidene fluoride membranes (Immobilon P, Millipore) and incubated with the indicated antibodies. Immunoreactive proteins were visualized with anti-rabbit or anti-mouse IgG conjugated to horseradish peroxidase (GE Healthcare), followed by processing with an ECL detection system (GE Healthcare) and imaging using a ChmiDoc system (Bio-Rad, Richmond, CA). Intensities of bands were quantitated by using Image J software.

### Nuclear protein extraction

Cells were washed with PBS and collected by centrifugation at 1,300 × *g* for 3 min. The cell pellet was lysed in hypotonic cytosol extraction buffer (10 mM HEPES, pH 7.9, 1.5 mM MgCl_2_, 10 mM KCl, 1.5 mM dithiothreitol (DTT), 0.05% Nonidet P-40, and a protease inhibitor mixture) for 15 min at 4 °C. Nuclei were pelleted by centrifugation at 15,000 rpm for 1 min at 4 °C and resuspended in nuclear extraction buffer (20 mM HEPES, pH 7.9, 1.5 mM MgCl_2_, 420 mM NaCl, 25% glycerol, 0.2 mM EDTA, 0.5 mM DTT, and a protease inhibitor mixture). After 20 min, the suspension was centrifuged at 15,000 rpm for 5 min at 4 °C, and the supernatant was collected as the nuclear protein extract.

### Real-time PCR analysis

Total RNA was isolated from cells using Trizol reagent (Life Technologies). cDNA was synthesized using Prime Script II (Takara Bio, Shiga, Japan). Quantitative real-time (q)PCR analysis was performed on a 7300 Fast Real-Time PCR system (Life Technologies) using FastStart Universal SYBR Green Master (Roche, Basel, Switzerland). All measurements were performed in triplicate. mRNA expression was normalized to glyceraldehyde-3-phosphate dehydrogenase (GAPDH) expression. Primers used to amplify specific genes were as follows: Spi-B1, forward 5´-CTC TGA ACC ACC ATG CTT GCT-3´ and reverse: 5´-TCC TTC TGG GTA CAA ACA GCT TAA-3´; GAPDH, forward 5´-ACC ATG TAG TTG AGG TCA ATG AAG G-3´ and reverse 5´-GGT GAA GGT CGG TGT GAA CG-3´.

## Additional Information

**How to cite this article**: Shinzawa, M. *et al*. Catalytic subunits of the phosphatase calcineurin interact with NF-κB-inducing kinase (NIK) and attenuate NIK-dependent gene expression. *Sci. Rep*. **5**, 10758; doi: 10.1038/srep10758 (2015).

## Supplementary Material

Supplementary Information

## Figures and Tables

**Table 1:** Genes identified as NIK-binding protein candidates.

Gene symbol	Gene name	IP
Anp32b	Acidic nuclear phosphoprotein 32 family, member B	ND
Dlg7	Discs, large homology 7	ND
Jun	Jun oncogene	ND
Jund	Jun proto-oncogene related gene d	ND
Lmnb1	Lamin B1	ND
Ldb1	LIM domain binding 1	ND
Phf8	PHD finger protein 8	ND
EG627352	Predicted gene	ND
CnAa	Calcineurin, catalytic subunit, alpha isoform	+
Arhgap12	Rho GTPase activating protein 12	−
Rnuxa	RNA U, small nuclear RNA export adaptor	−
Sdccag8	Serologycally defined colon cancer antigen 8	ND
Snrpf	Small nuclear ribonucleoprotein polypeptide F	ND
Slc46a2	Solute carrier family 46, member 2	ND
Svil	Supervillin	ND
Ubp1	Upstream binding protein	ND
Atl3	Atlastin GTPase 3	+/−
Col4a1	Collagen, type IV, alpha 1	ND
Dync1li2	Dynein, cytoplasmin 1 light intermediated chain 2	−
Exosc8	Exosome component 8	ND
Faf1	Fas-associated factor 1	ND
Hnrnpr	Heterogeneous nuclear ribonucleoprotein R	−
Hspa8	Heat shock protein 8	ND
LOC100042644	similar to ribosomal protein L39	ND
Ndufa3	NADH dehydrogenase (ubiquinone) 1 alpha subcomplex, 3	ND
Nkap	NFKB activating protein	ND
Rpl4	Ribosomal protein L4	ND
Srrm1	Serine/Arginine repetitive matrix 1	ND
Syncrip	Synaptotagmin binding, cytoplasmic RNA interacting protein	ND

Column of IP shows results of immunoprecipitation experiment. “+” indicates that interaction was confirmed. “−” indicates that interaction was not detected. ND indicates that verifications have not been completed yet.

## References

[b1] Vallabhapurapu S. & Karin M. Regulation and function of NF-kappaB transcription factors in the immune system. Annu. Rev. Immunol. 27, 693–733 (2009). 19302050 10.1146/annurev.immunol.021908.132641

[b2] Inoue J., Gohda J., Akiyama T. & Semba K. NF-kappaB activation in development and progression of cancer. Cancer Sci. 98, 268–274 (2007). 17270016 10.1111/j.1349-7006.2007.00389.x PMC11158158

[b3] Hoffmann A., Natoli G. & Ghosh G. Transcriptional regulation via the NF-kappaB signaling module. Oncogene 25, 6706–6716 (2006). 17072323 10.1038/sj.onc.1209933

[b4] Oeckinghaus A., Hayden M. S. & Ghosh S. Crosstalk in NF-κB signaling pathways. Nat. Immunol. 12, 695–708 (2011). 21772278 10.1038/ni.2065

[b5] Sun S. C. The noncanonical NF-κB pathway. Immunol. Rev. 246, 125–140 (2012). 22435551 10.1111/j.1600-065X.2011.01088.x PMC3313452

[b6] Malinin N. L., Boldin M. P., Kovalenko A. V. & Wallach D. MAP3K-related kinase involved in NF-kappaB induction by TNF, CD95 and IL-1. Nature 385, 540–544 (1997). 9020361 10.1038/385540a0

[b7] Miyawaki S. . A new mutation, aly, that induces a generalized lack of lymph nodes accompanied by immunodeficiency in mice. Eur. J. Immunol. 24, 429–434 (1994). 8299692 10.1002/eji.1830240224

[b8] Shinkura R. . Alymphoplasia is caused by a point mutation in the mouse gene encoding Nf-kappa b-inducing kinase. Nat. Genet. 22, 74–77 (1999). 10319865 10.1038/8780

[b9] Shinzawa M. . Splenic extramedullary hemopoiesis caused by a dysfunctional mutation in the NF-κB-inducing kinase gene. Biochem. Biophys. Res. Commun. 414, 773–778 (2011). 22005462 10.1016/j.bbrc.2011.10.001

[b10] Gerondakis S. . Unravelling the complexities of the NF-kappaB signalling pathway using mouse knockout and transgenic models. Oncogene 25, 6781–6799 (2006). 17072328 10.1038/sj.onc.1209944

[b11] Xiao G., Harhaj E. W. & Sun S. C. NF-kappaB-inducing kinase regulates the processing of NF-kappaB2 p100. Mol. Cell. 7, 401–409 (2001). 11239468 10.1016/s1097-2765(01)00187-3

[b12] Annunziata C. M. . Frequent engagement of the classical and alternative NF-kappaB pathways by diverse genetic abnormalities in multiple myeloma. Cancer Cell 12, 115–130 (2007). 17692804 10.1016/j.ccr.2007.07.004 PMC2730509

[b13] Keats J. J. . Promiscuous mutations activate the noncanonical NF-kappaB pathway in multiple myeloma. Cancer Cell 12, 131–144 (2007). 17692805 10.1016/j.ccr.2007.07.003 PMC2083698

[b14] Enzler T. . Alternative and classical NF-kappa B signaling retain autoreactive B cells in the splenic marginal zone and result in lupus-like disease. Immunity 25, 403–415 (2006). 16973390 10.1016/j.immuni.2006.07.010

[b15] Thu Y. M. & Richmond A. NF-κB inducing kinase: a key regulator in the immune system and in cancer. Cytokine Growth Factor Rev. 21, 213–226 (2010). 20685151 10.1016/j.cytogfr.2010.06.002 PMC2939163

[b16] Zarnegar B. J. . Noncanonical NF-kappaB activation requires coordinated assembly of a regulatory complex of the adaptors cIAP1, cIAP2, TRAF2 and TRAF3 and the kinase NIK. Nat. Immunol. 9, 1371–1378 (2008). 18997794 10.1038/ni.1676 PMC2676931

[b17] Lin X. . Molecular determinants of NF-kappaB-inducing kinase action. Mol. Cell. Biol. 18, 5899–5907 (1998). 9742107 10.1128/mcb.18.10.5899 PMC109176

[b18] Razani B. . Negative feedback in noncanonical NF-kappaB signaling modulates NIK stability through IKKalpha-mediated phosphorylation. *Sci*. Signal. 3, ra41 (2010). 10.1126/scisignal.2000778 PMC2913610 20501937

[b19] Li H., Rao A. & Hogan P. G. Interaction of calcineurin with substrates and targeting proteins. Trends Cell. Biol. 21, 91–103 (2011). 21115349 10.1016/j.tcb.2010.09.011 PMC3244350

[b20] Palkowitsch L. . The Ca2+ -dependent phosphatase calcineurin controls the formation of the Carma1-Bcl10-Malt1 complex during T cell receptor-induced NF-kappaB activation. J. Biol. Chem. 286, 7522–7534 (2011). 21199863 10.1074/jbc.M110.155895 PMC3045007

[b21] Frischbutter S., Gabriel C., Bendfeldt H., Radbruch A. & Baumgrass R. Dephosphorylation of Bcl-10 by calcineurin is essential for canonical NF-κB activation in Th cells. Eur. J. Immunol. 41, 2349–2357 (2011). 21674474 10.1002/eji.201041052

[b22] Nemoto N., Miyamoto-Sato E., Husimi Y., Yanagawa H. *In vitro* virus” Bonding of mRNA bearing puromycin at the 3’-terminal end to the C-terminal end of its encoded protein on the ribosome *in vitro*. FEBS Lett. 414, 405–408 (1997). 9315729 10.1016/s0014-5793(97)01026-0

[b23] Miyamoto-Sato E. . Cell-free cotranslation and selection using *in vitro* virus for high-throughput analysis of protein-protein interactions and complexes. Genome Res. 15, 710–717 (2005). 15867431 10.1101/gr.3510505 PMC1088299

[b24] Akiyama T. . Mitochondria-nucleus shuttling FK506-binding protein 51 interacts with TRAF proteins and facilitates the RIG-I-like receptor-mediated expression of type I IFN. PLoS One 9, e95992 (2014). 24788966 10.1371/journal.pone.0095992 PMC4006813

[b25] Rusnak F. & Mertz P. Calcineurin: form and function. Physiol. Rev. 80, 1483–1521 (2000). 11015619 10.1152/physrev.2000.80.4.1483

[b26] Ngo V. N. . Lymphotoxin alpha/beta and tumor necrosis factor are required for stromal cell expression of homing chemokines in B and T cell areas of the spleen. J. Exp. Med. 189, 403–412 (1999). 9892622 10.1084/jem.189.2.403 PMC2192983

[b27] Cyster J. G. . Follicular stromal cells and lymphocyte homing to follicles. Immunol. Rev. 176, 181–193 (2000). 11043777 10.1034/j.1600-065x.2000.00618.x

[b28] Chin R. K. . Lymphotoxin pathway directs thymic Aire expression. Nat. Immunol. 4, 1121–1127 (2003). 14517552 10.1038/ni982

[b29] Britanova L. V., Makeev V. J. & Kuprash D. V. *In vitro* selection of optimal RelB/p52 DNA-binding motifs. Biochem. Biophys. Res. Commun. 365, 583–588 (2008). 17996728 10.1016/j.bbrc.2007.10.200

[b30] Lovas A. . Differential RelA- and RelB-dependent gene transcription in LTbetaR-stimulated mouse embryonic fibroblasts. BMC Genomics 9, 606 (2008). 19087315 10.1186/1471-2164-9-606 PMC2637282

[b31] Akiyama N. . Limitation of immune tolerance–inducing thymic epithelial cell development by Spi-B–mediated negative feedback regulation J. Exp. Med. 211, 2425–2438 (2014). 25385757 10.1084/jem.20141207 PMC4235644

[b32] Akiyama T., Shinzawa M. & Akiyama N. TNF receptor family signaling in the development and functions of medullary thymic epithelial cells. Front. Immunol. 3, 278 (2012). 22969770 10.3389/fimmu.2012.00278 PMC3432834

[b33] Matsushima A. . Essential role of nuclear factor (NF)-kappaB-inducing kinase and inhibitor of kappaB (IkappaB) kinase alpha in NF-kappaB activation through lymphotoxin beta receptor, but not through tumor necrosis factor receptor I. J. Exp. Med. 193, 631–636 (2001). 11238593 10.1084/jem.193.5.631 PMC2193391

[b34] Dejardin E. . The lymphotoxin-beta receptor induces different patterns of gene expression via two NF-kappaB pathways. Immunity 17, 525–535 (2002). 12387745 10.1016/s1074-7613(02)00423-5

[b35] Yamaguchi N., Oyama M., Kozuka-Hata H. & Inoue J. Involvement of A20 in the molecular switch that activates the non-canonical NF-кB pathway. Sci. Rep. 3, 2568 (2013). 10.1038/srep02568 PMC3764444 24008839

[b36] Saitoh T. . TWEAK induces NF-kappaB2 p100 processing and long lasting NF-kappaB activation. J. Biol. Chem. 278, 36005–36012 (2003). 12840022 10.1074/jbc.M304266200

[b37] Feske S. Calcium signalling in lymphocyte activation and disease. Nat. Rev. Immunol. 7, 690–702 (2007). 17703229 10.1038/nri2152

[b38] Kang Y. J. . Calcineurin negatively regulates TLR-mediated activation pathways. J. Immunol. 179, 4598–4607 (2007). 17878357 10.4049/jimmunol.179.7.4598

[b39] Morris G. P. & Allen P. M. How the TCR balances sensitivity and specificity for the recognition of self and pathogens. Nat. Immunol. 13, 121–128 (2012). 22261968 10.1038/ni.2190 PMC13052442

[b40] Miyamoto-Sato E. . A comprehensive resource of interacting protein regions for refining human transcription factor networks. PLoS One 5, e9289 (2010). 20195357 10.1371/journal.pone.0009289 PMC2827538

