# Supplementary material for: Catalytic subunits of the phosphatase calcineurin interact with NF-κB-inducing kinase (NIK) and attenuate NIK-dependent gene expression
Source: Sci Rep. 2015 Jun 1;5:10758. doi: 10.1038/srep10758 (PMC5377069; doi:10.1038/srep10758)
Supplement: Supplementary Information [file srep10758-s1.pdf]

Supplementary Figure and legends

Title: Catalytic subunits of the phosphatase calcineurin interact with NF-κB-inducing kinase (NIK) and attenuate NIK-dependent gene expression

Authors: Miho Shinzawa, Hiroyasu Konno, Junwen Qin, Nobuko Akiyama, Maki Miyauchi, Hiroyuki Ohashi, Etsuko Miyamoto-Sato, Hiroshi Yanagawa, Taishin Akiyama and Jun-ichiro Inoue

Supplementary Figure 1

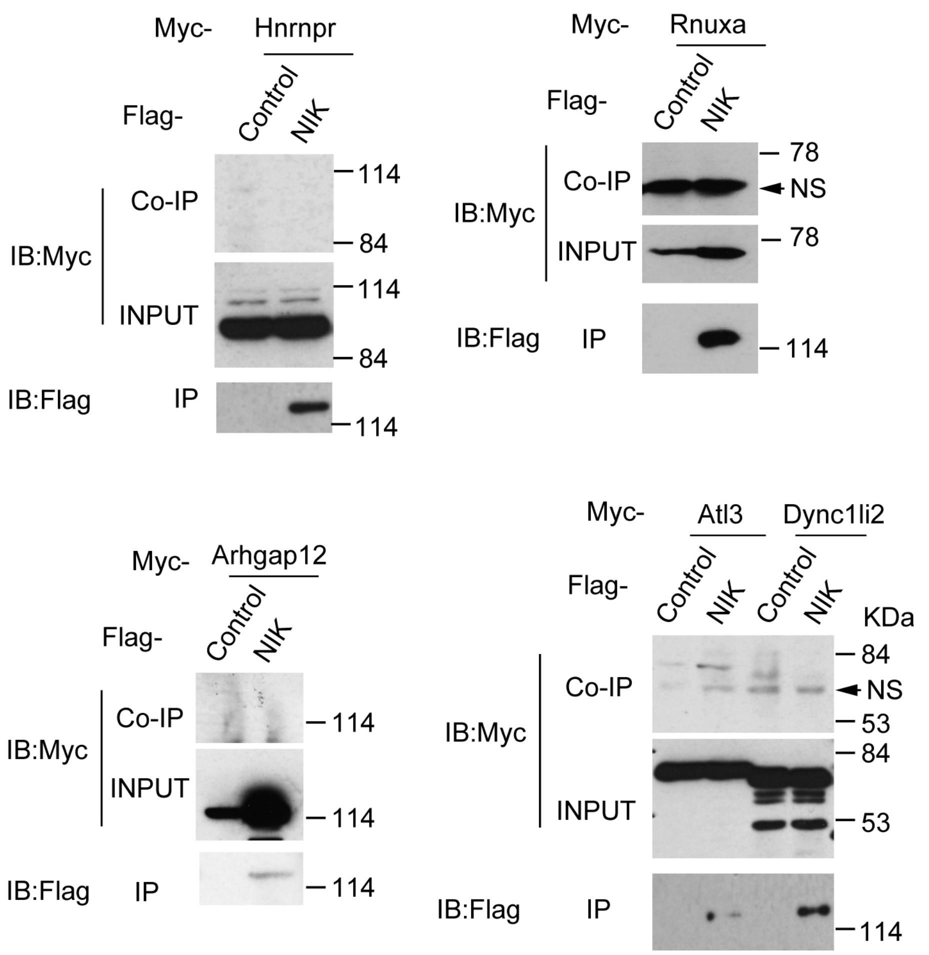

Supplementary Figure 1. Co-immunoprecipitation of NIK with candidate proteins. Flag-tagged NIK and Myc-tagged candidates (Hnrnpr, Rnuxa, Arhgap12, Atl3, and Dync1li1) were expressed in HEK293T cells. Control indicates the Flag-tagged expression vector. The upper panel (Co-IP) shows western blotting of immunoprecipitates using an anti-Flag antibody to detect co-immunoprecipitation of Myc-tagged CnAα or CnAβ. The middle panel shows western blotting of total cell lysates using an anti-Myc antibody. The lower panels show western blotting of immunoprecipitates using the anti-Flag antibody to detect Flag-tagged NIK. NS indicate bands of IgG chains used for immunoprecipitation. Blots are cropped for clarity.

Supplementary Figure 2

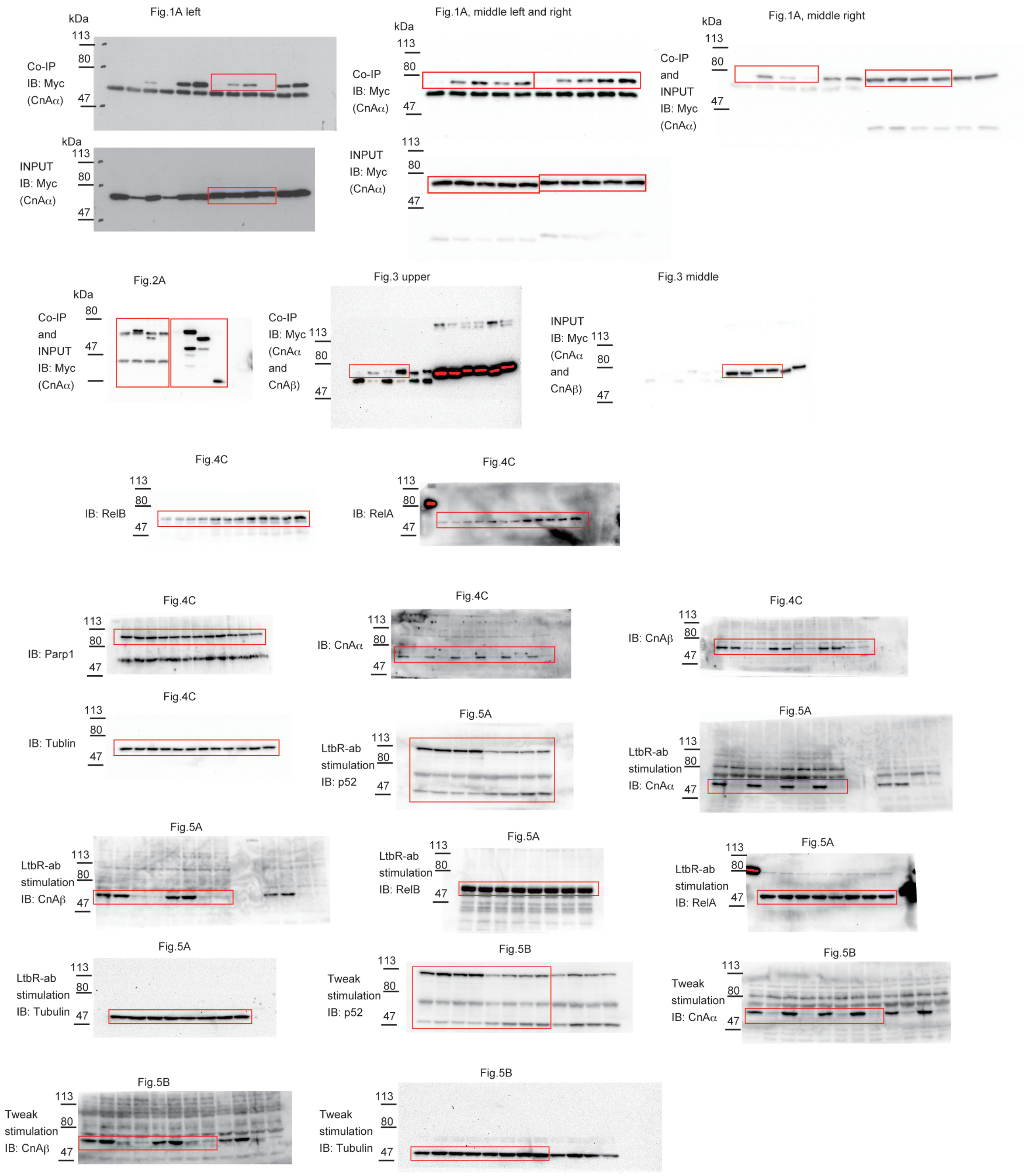

Supplementary Figure 2. Full-length blots of key data.

Red rectangles show cropped regions.
